# Areas of Interest and Attitudes Toward Antiobesity Drugs: Thematic and Quantitative Analysis Using Twitter

**DOI:** 10.2196/24336

**Published:** 2021-10-26

**Authors:** Miguel Angel Alvarez-Mon, Maria Llavero-Valero, Angel Asunsolo del Barco, Cristina Zaragozá, Miguel A Ortega, Guillermo Lahera, Javier Quintero, Melchor Alvarez-Mon

**Affiliations:** 1 Department of Psychiatry and Mental Health Hospital Universitario Infanta Leonor Madrid Spain; 2 Department of Medicine and Medical Specialities Faculty of Medicine and Health Sciences University of Alcala Alcalá de Henares Spain; 3 Department of Endocrinology and Clinical Nutrition Hospital Universitario Infanta Leonor Madrid Spain; 4 Department of Preventive Medicine and Public Health School of Medicine University of Navarra Pamplona Spain; 5 Department of Surgery, Medical and Social Sciences Faculty of Medicine and Health Sciences University of Alcala Madrid Spain; 6 Ramón y Cajal Institute of Sanitary Research Madrid Spain; 7 Pharmacology Unit, Biomedical Sciences Department University of Alcalá Madrid Spain; 8 Centro de Investigación Biomédica en Red de Salud Mental Madrid Spain; 9 Psychiatry Service Hospital Universitario Principe de Asturias Alcalá de Henares Spain; 10 Department of Legal Medicine and Psychiatry Complutense University Madrid Spain; 11 Internal Medicine and Autoimmunity/Rheumatology Service Hospital Universitario Principe de Asturias Madrid Spain; 12 Centro de Investigación Biomédica en Red en Enfermedades Hepáticas y Digestivas Madrid Spain

**Keywords:** obesity, social media, Twitter, drug therapy, pharmacotherapy, attitude, thematic analysis, quantitative analysis, drug

## Abstract

**Background:**

Antiobesity drugs are prescribed for the treatment of obesity in conjunction with healthy eating, physical activity, and behavior modification. However, poor adherence rates have been reported. Attitudes or beliefs toward medications are important to ascertain because they may be associated with patient behavior. The analysis of tweets has become a tool for health research.

**Objective:**

The aim of this study is to investigate the content and key metrics of tweets referring to antiobesity drugs.

**Methods:**

In this observational quantitative and qualitative study, we focused on tweets containing hashtags related to antiobesity drugs between September 20, 2019, and October 31, 2019. Tweets were first classified according to whether they described medical issues or not. Tweets with medical content were classified according to the topic they referred to: side effects, efficacy, or adherence. We additionally rated it as positive or negative. Furthermore, we classified any links included within a tweet as either scientific or nonscientific. Finally, the number of retweets generated as well as the dissemination and sentiment score obtained by the antiobesity drugs analyzed were also measured.

**Results:**

We analyzed a total of 2045 tweets, 945 of which were excluded according to the criteria of the study. Finally, 320 out of the 1,100 remaining tweets were also excluded because their content, although related to drugs for obesity treatment, did not address the efficacy, side effects, or adherence to medication. Liraglutide and semaglutide accumulated the majority of tweets (682/780, 87.4%). Notably, the content that generated the highest frequency of tweets was related to treatment efficacy, with liraglutide-, semaglutide-, and lorcaserin-related tweets accumulating the highest proportion of positive consideration. We found the highest percentages of tweets with scientific links in those posts related to liraglutide and semaglutide. Semaglutide-related tweets obtained the highest probability of likes and were the most disseminated within the Twitter community.

**Conclusions:**

This analysis of posted tweets related to antiobesity drugs shows that the interest, beliefs, and experiences regarding these pharmacological treatments are heterogeneous. The efficacy of the treatment accounts for the majority of interest among Twitter users.

## Introduction

Obesity is an increasingly prevalent disease, with high rates of associated morbidity and mortality [1]. The treatment of obesity remains only partially effective [2]. Moreover, the pharmacological treatment of obesity is becoming a more significant tool in the management of the disease [2]. However, frequency in the use of antiobesity drug treatments among those patients that could potentially benefit from them is minimal [3]. Moreover, both personal and social attitudes, combined with accessible information on available treatments, have been shown to be relevant for obtaining the expected clinical outcomes of pharmacological intervention [4]. Furthermore, it has been shown that social support is a potential beneficial component of weight loss programs [5,6].

In recent years, social media has become a pivotal instrument for disseminating knowledge [7]. Accordingly, the internet has modified how people communicate and how they share and seek out information regarding health [8]. Social networks are extensively used for the study of obesity, including the analysis of public attitudes, the social support of patients, patients’ behavior, and treatment efficacy [9-11]. Being that information pertaining to health posted over social media is oftentimes more spontaneous in nature, it serves more as a complementary perspective to data collected from medical surveys, clinical trials, and consultancies with medical professionals [12-14].

Twitter, one of the most popular and widely used social media platforms, is currently considered to be an effective channel of communication [15]. Within this context, different agents in the health sector have realized Twitter´s potential for acquiring and distributing medical information [16,17]. In addition, Twitter users demonstrate a great interest in obesity and eating disorders [18,19]. Moreover, it has been shown that Twitter can be an effective platform for delivering interventions aimed at treating obesity [20]. The analysis of tweets about obesity, diet, and treatments is a recent relevant area of study for understanding the actual sentiments of society, patients, and health providers [21]. The trivialization, stigmatization, and mockery directed at obesity and other disorders by Twitter users have been reported [22]. Until now, however, topics of interest among Twitter users regarding the pharmacological treatment of obesity have not been identified. Furthermore, the dissemination of medication-related tweets tied to obesity remains unknown. The analysis of the feelings and experiences toward pharmacological treatment is relevant for the understanding of patients’ attitudes to these drugs and the identification of concerns and needs potentially related to treatment adherence [23,24].

In this study, we performed an analysis of the content and key metrics of all the tweets generated concerning medications approved by the US Food and Drug Administration (FDA) for obesity treatment over a period of 6 weeks. We also investigated the areas of interest of those tweets containing medical content and the inclusion of links to related informative resources. Finally, we investigated the tweets’ dissemination and overall sentiment.

## Methods

### Data Collection

In this observational quantitative and qualitative study, we focused on searching for tweets that referred to medications approved by the FDA for the treatment of overweight or obese status: Xenical, orlistat, Alli, Belviq, lorcaserin, Qsymia, phentermine-topiramate, Contrave, bupropion-naltrexone, Saxenda, Victoza, liraglutide, Ozempic, and semaglutide. The inclusion criteria for tweets were the following: being public; using any of the previously mentioned hashtags; being posted between September 20, 2019, and October 31, 2019; and being posted in English language. This 6-week period was chosen to avoid any potential bias within the content of the tweets. In addition, we obtained the number of likes each tweet generated, the date and time of each tweet, and the potential reach and impact of each hashtag.

### Search Tool

We used the Twitter Firehose data stream, which is managed by Gnip and allows access to 100% of all public tweets that match a certain criteria (query) [25]. In our study, the search criteria were the previously mentioned hashtags. Tweet Binder, the search engine we employed, uses node.js and PHP language, which enabled us to analyze tweets in JavaScript Object Notation (JSON) format (used by Gnip).

### Content Analysis Process

All 2045 retrieved tweets were directly inspected by 2 raters (MAAM and MLV). First, we scanned all of the tweets, excluding 945 tweets that provided information that was too limited (eg, tweets consisting mainly of hashtags), that contained only pictures, or that included hashtags of more than 1 treatment. All the remaining tweets were considered for thematic content analysis. Second, we created a codebook based on our research questions, our previous experience in analyzing tweets, and what we determined to be the most common tweet themes. Third, 2 raters analyzed 150 tweets separately to test the suitability of the codebook. Discrepancies were discussed between the raters and with another author (MAM). After the codebook was revised, the interrater reliability was reassessed with a different set of 150 tweets. As this resulted in adequate κ values (range 0.68-0.99), the raters then proceeded to analyze 1100 tweets equally distributed among both. Each tweet, depending on its content, was categorized under side effects, efficacy, or adherence. In addition, users were classified into 3 categories: patients and relatives, health professionals, or health institutions. These categorizations were defined based on the description of user profiles and the content of user tweets. In those cases in which the nature of the user was not possible to know, they were classified as indeterminate. Finally, we analyzed any links included within a tweet, classifying them as either scientific or nonscientific. More specifically, those links attributed to a scientific source, including medical journals, academic institutions, hospitals, and official websites, were classified as scientific. The classification criteria we used and examples of tweets are shown in Textbox 1**.**

**Examples of tweets related to efficacy, side effects, and adherence (usernames and personal names have been removed).**

**Efficacy** (**the ability or inability of a treatment to provide a beneficial effect)**

- “Oral semaglutide can effectively and safely reduce blood glucose, body weight and systolic blood pressure: A systematic review and meta-analysis.”
- “Ozempic is superior to Invokana in reducing A1c and body weight”.
- “More than just #weightloss ? Liraglutide improves hepatic steatosis and metabolic dysfunctions in a 3-week dietary mouse model of nonalcoholic steatohepatitis.”
- “The PIONEER 4 trial showed that oral semaglutide is noninferior to injectable liraglutide and superior to placebo in improving glycemic control and weight loss at 26 weeks among patients with type 2 #diabetes.”.

**Side effects (any effect that is secondary to the one intended either adverse or beneficial; tweets discussing tolerability of the drug were also included)**

- “No side effects with the Ozempic and I’ve been on it since July of last year. Now the metformin is a whole different story. First week or so the sight of food made me sick and it made my stomach act up. I’m actually glad to be off of it.”
- “Does Contrave Make Anyone Else's Hands Shake?”
- “I saw the Saxenda results on people I know. It is fantastic. However, it comes with its challenges. Nausea, headaches and terrible moods. glucagon-like peptide 1 (GLP-1) analogs, such as liraglutide, is the possibility of developing pancreatitis. #usmle”
- “A bit personal, but I'm curious to hear others' stories. I've been taking Ozempic for a year or so, but the last 6-8 weeks I've started throwing up quite a bit. I don't have a history of this at all. Anyone else had this problem with Ozempic? Thank you!”
- “Those embalming leaves seem to have less side effects than ozempic.”
- “Gallstone Disease in Patients Treated with Liraglutide: In a large randomized trial a small but statistically significant rise in this adverse event was noted.”

**Adherence** (**the degree of conformity to the recommendations about the treatment with respect to the timing, dosage, or frequency)**

- “I'm a quibbler, I can't help it. There is a medicine called Qsymia that seems effective for long term weight loss. Its not without side effects or risks.”
- “Morning Twitter nightmare week over. Week 21 liraglutide. Despite everything total adherence. Adherence to duloxetine not good. Quorn burger for breakfast & fruit. Happy today.”

### Measuring Interest and Influence on Twitter

We analyzed the number of likes generated by each tweet as an indicator of user interest on a given topic. We also measured the potential reach and impact of all analyzed hashtags in order to best assess tendencies in the dissemination of tweets. In this study, impact was defined as a numerical value representing the potential views a tweet may receive, while reach was defined as a numerical value measuring the potential audience of the hashtag.

In addition, we measured how positive or negative a hashtag was on a scale from 1 (negative) to 100 (positive). Sentiment analysis tools were used to analyze all words contained in a tweet, with each word having its own score that could vary depending on the context. The average score of all the tweets with a certain hashtag determined that hashtag’s overall sentiment score. According to this score, we classified each hashtag into 3 categories: negative (0-40), neutral (>40-60), and positive (>60-100).

### Ethical Considerations

This study was approved by the Research Ethics Committee at the University of Alcala.

### Statistical Analysis

A descriptive study of the sample was performed. The qualitative variables are described as absolute frequency (number) and relative frequency (percentage). The percentages found were compared using the chi-square test. The numbers of retweets and likes per original tweet about the different hashtags were verified by graphs and statistical test (Kolmogorov-Smirnof test), and they did not follow a normal distribution. The differences found between the treatment groups were compared using the Kruskal-Wallis test. All statistical analyses were performed using SPSS version 25 (IBM Corp).

## Results

### Liraglutide and Semaglutide Accumulated the Most Interest Among Twitter Users

Our search tool provided a total of 2045 tweets, 945 of which were excluded according to the criteria of the study. Next, 320 out of the 1100 remaining tweets were also excluded because their content, although related to drugs for obesity treatment, did not address the efficacy, side effects, or adherence to the medication. Finally, we classified the content of the remaining 780 tweets.

The number of tweets with hashtags referencing the 6 drug groups approved for obesity treatment were significantly different, with the incidence of tweets related to liraglutide and semaglutide at least being 10 times higher than that related to the other 5 drug groups (Table 1).

Next, we found significant differences in the distribution of the content. Notably, the content that generated the highest frequency of tweets was that related to treatment efficacy, with liraglutide-, semaglutide-, and lorcaserin-related tweets accumulating the highest proportion of positive consideration (*P*<.001). In contrast, the highest percentage of tweets with a negative valuation towards efficacy was found in those posts related to bupropion-naltrexone (3/30, 10%), while those containing a mention of liraglutide (14/319, 4.4%) and semaglutide (2/363, 0.6%) had a much lower negative percentage.

Tweets with a negative valuation of side effects were mainly observed in those related to orlistat (4/15, 26.7%) and bupropion-naltrexone (4/30, 13.3%) but rarely in those mentioning liraglutide, semaglutide, and phentermine-topiramate. On the other hand, tweets with a positive valuation of side effects were minimal and were found mainly in those posts related to bupropion-naltrexone, semaglutide, and liraglutide (*P*=.04). Finally, the frequency of tweets with content related to adherence to treatment was low, with negative considerations predominant among liraglutide, bupropion-naltrexone, and semaglutide (*P*<.001). On the other hand, positive valuations of adherence to treatment were observed in those tweets related to orlistat, semaglutide, and liraglutide.

**Table 1:** Descriptive characteristics of the original tweets included in the analysis categorized by total amount per drug and category (side effects, efficacy, adherence, and link).

Category		Orlistat, n (%) (N=15)	Lorcaserin, n (%) (N=27)	Phentermine-topiramate, n (%) (N=26)	Bupropion-naltrexone, n (%) (N=30)	Liraglutide, n (%) (N=319)	Semaglutide, n (%) (N=363)	*P* value^a^
**Side effects**								.04
	No mention	11 (73.3)	27 (100)	25 (96.2)	25 (83.3)	299 (93.7)	337 (92.8)	
	Positive	0 (0)	0 (0)	0 (0)	1 (3.3)	2 (0.6)	8 (2.2)	
	Negative	4 (26.7)	0 (0)	1 (3.8)	4 (13.3)	18 (5.6)	18 (5.0)	
**Efficacy**								<.001
	No mention	9 (60.0)	8 (29.6)	15 (57.7)	14 (46.7)	62 (19.4)	131 (36.1)	
	Positive	6 (40.0)	19 (70.4)	11 (42.3)	13 (43.3)	243 (76.2)	230 (63.4)	
	Negative	0 (0)	0 (0)	0 (0)	3 (10.0)	14 (4.4)	2 (0.6)	
**Adherence**								<.001
	No mention	14 (93.3)	27 (100)	26 (100)	28 (93.3)	288 (90.3)	336 (92.6)	
	Positive	1 (6.7)	0 (0)	0 (0)	0 (0)	3 (0.9)	21 (5.8)	
	Negative	0 (0)	0 (0)	0 (0)	2 (6.7)	28 (8.8)	6 (1.7)	
**Link**								<.001
	None	6 (40.0)	2 (7.4)	4 (15.4)	18 (60.0)	93 (29.2)	91 (25.1)	
	Scientific	1 (6.7)	11 (40.7)	5 (19.2)	0 (0)	196 (61.4)	270 (74.4)	
	Nonscientific	8 (53.3)	14 (51.9)	17 (65.4)	12 (40)	30 (9.4)	2 (0.6)	

^a^Chi-square tests were conducted to assess statistical differences.

**Scientific Links Were Mainly Found Within Liraglutide- and Semaglutide-Related Tweets**

We found significant differences between the distribution of those tweets including a link, whether scientific or nonscientific, among the 6 different drug groups (*P*<.001 Table 1). More specifically, we found the highest percentages of tweets with scientific links in those posts related to liraglutide (196/319, 61.4%) and semaglutide (270/363, 74.4%), followed by those tweets referencing lorcaserin (11/27, 40.7%) and phentermine-topiramate (5/26, 19.2%). The frequency of tweets with a nonscientific link was very low in those related to semaglutide and liraglutide; on the other hand, more than half of the tweets referencing phentermine-topiramate, lorcaserin, and orlistat included a nonscientific link.

As liraglutide and semaglutide accumulated the majority of tweets (682/780, 87.4%), we decided to investigate the use of links in these tweets depending on their content (Table 2). The use of links was mainly focused on those tweets with a positive consideration towards the efficacy of the treatment, whereas in those tweets referencing side effects and adherence to treatment, the use of links was marginal.

**Table 2:** Descriptive characteristics of the original tweets included in the analysis categorized by total amount per drug and category.

Category		Liraglutide, n (%)		*P* value^a^	Semaglutide, n (%)		*P* value^a^
		Without link (N=93)	With link (N=226)		Without link (N=91)	With link (N=272)	
**Side effects**				.006			<.001
	No mention	81 (87.1)	218 (96.5)		77 (84.6)	260 (95.6)	
	Positive	1 (1.1)	0 (0)		2 (2.2)	6 (2.2)	
	Negative	11 (11.8)	8 (3.5)		12 (13.2)	6 (2.2)	
**Efficacy**				<.001			<.001
	No mention	24 (25.8)	38 (16.8)		17 (18.7)	114 (41.9)	
	Positive	55 (59.1)	188 (83.2)		72 (79.1)	158 (58.1)	
	Negative	14 (15.1)	0 (0)		2 (2.2)	0 (0)	
**Adherence**				<.001			<.001
	No mention	73 (78.5)	215 (95.1)		80 (87.9)	256 (94.1)	
	Positive	3 (3.2)	0 (0)		5 (5.5)	16 (5.9)	
	Negative	17 (18.3)	11 (4.9)		6 (6.6)	0 (0)	

^a^Chi-square tests were conducted to assess statistical differences.

### Semaglutide-Related Tweets Obtained the Highest Probability of Likes and Were the Most Disseminated Within the Twitter Community

We found that the probabilities of a tweet being liked between the groups were significantly different (*P*<.001). Semaglutide-related tweets accumulated the highest number of likes per tweet (median 3; 95% CI 1-12). In addition, we analyzed the number of likes received per tweet as classified by the inclusion or absence of a link. We found no differences in the median of likes per tweet between those posts including or not including a link (*P*=.27).

We found that semaglutide-related tweets had the highest potential reach and impact (2,522,621 and 4,676,763 , respectively), which was double that of liraglutide (719,382 and 1,631,062, respectively). On the other hand, both parameters were markedly lower for bupropion-naltrexone (996,398 and 1,603,556, respectively), orlistat (486,533 and 697,956 , respectively), phentermine-topiramate (183,919 and 187,094, respectively), and lorcaserin (29,420 and 30,341, respectively).

Regarding the sentiment analyses of the content of the tweets, we found that those posts related to semaglutide (mean 79.67), liraglutide (mean 61.46), lorcaserin (mean 75.14), and phentermine-topiramate (mean 60.06) received positive sentiment. However, the sentiment was neutral for orlistat (mean 43.9) and bupropion-naltrexone (mean 53.8).

### Health Institutions Were the Most Active Twitter Users

We investigated the type of users that posted the tweets. Of the total number of tweets, 7.9% (62/780) were posted by users identified as patients or relatives, 16% (125/780) by health institutions, and 27.1% (211/780) by health care professionals. Of the remaining 49% (382/780) of tweets, the users were considered indeterminate.

Next, we investigated those tweets related to side effects according to the different types of users and found significant differences in the frequency and content of the postings (Table 3; *P*<.001). Patients were the users that posted most about the presence of side effects, whereas health institutions mentioned the presence of side effects the least. Moreover, we also found significant differences between users in regards to tweets about efficacy (*P*<.001) and adherence (*P*<.001). Interestingly, patients were also those who most frequently expressed a lack of efficacy or adherence (Table 4 and Table 5). On the other hand, users classified as health institutions were those that posted most frequently on the efficacy of treatment and promoted adherence to it. Additionally, we assessed who the users were that most frequently included a link within their tweets (Table 6). We found that health institutions included a link, either designated as scientific or nonscientific, more frequently in their posts than did users classified as health professionals or patients (*P*<.001). Finally, we assessed the frequency of user postings according to the different antiobesity drugs analyzed, finding significant differences among them. In particular, health institutions generated the majority of tweets concerning the latest antiobesity drugs.

**Table 3:** Descriptive characteristics of those tweets referencing side effects in which the user was categorized as a patient, health professional, or health institution. Tweets were further classified as either not mentioning side effects or mentioning them positively or negatively.

Side effects	Patients, n (%)	Health professionals, n (%)	Health institutions, n (%)	Total, n (%)
No mention	40 (11.59)	114 (33.04)	191 (55.36)	345 (100)
Positive	2 (66.67)	0 (0)	1 (33.33)	3 (100)
Negative	11 (39.29)	8 (28.57)	9 (32.14)	28 (100)
Total	53 (14.10)	122 (32.45)	201 (53.46)	376 (100)

**Table 4:** Descriptive characteristics of those tweets referencing efficacy in which the user was categorized as a patient, health professional, or health institution. Tweets were further classified as either not mentioning efficacy or mentioning it positively or negatively.

Efficacy	Patients, n (%)	Health professionals, n (%)	Health institutions, n (%)	Total, n (%)
No mention	27 (20.45)	34 (25.76)	71 (53.79)	132 (100)
Positive	19 (8.19)	83 (35.78	130 (56.03)	232 (100)
Negative	7 (58.33)	5 (41.67)	0 (0)	12 (100)
Total	53 (14.10)	122 (32.45)	201 (53.46)	376 (100)

**Table 5:** Descriptive characteristics of those tweets referencing adherence in which the user was categorized as a patient, health professional, or health institution. Tweets were further classified as either not mentioning adherence or mentioning it positively or negatively.

Adherence	Patients, n (%)	Health professionals, n (%)	Health institutions, n (%)	Total, n (%)
No mention	37 (10.60)	117 (33.52)	195 (55.87)	349 (100)
Positive	1 (12.5)	1 (12.5)	6 (75)	8 (100)
Negative	15 (78.95)	4 (21.05)	0 (0)	19 (100)
Total	53 (14.10)	122 (32.45)	201 (53.46)	376 (100)

**Table 6:** Descriptive characteristics of those tweets in which the user was categorized as a patient, health professional, or health institutions, and further designated as either not including a link or including a scientific or a nonscientific link.

Link	Patients, n (%)	Health professionals, n (%)	Health institutions, n (%)	Total, n (%)
None	52 (50)	39 (37.50)	13 (12.50)	104 (100)
Scientific	0 (0)	70 (31.25)	154 (68.75)	224 (100)
Nonscientific	1 (2.1)	13 (27.08)	34 (70.83)	48 (100)
Total	53 (14.10)	122 (32.45)	201 (53.46)	376 (100)

## Discussion

### Principal Findings

In this study, we have found that Twitter users show an interest in antiobesity drugs and mainly focus on semaglutide and liraglutide. These tweets are centered on the efficacy of the treatment and principally refer to liraglutide, semaglutide, and lorcaserin. Tweet content containing a negative consideration of side effects was mainly observed in those tweets related to orlistat and bupropion-naltrexone. The frequency of tweets with content related to adherence to treatment was marginal. The highest percentages of tweets with scientific links were observed in those related to liraglutide and semaglutide. Furthermore, those tweets referencing semaglutide obtained the highest potential reach and impact.

Diet, exercise, and lifestyle are considered relevant elements for maintaining a weight within the recommended range [26]. The prevention and treatment of overweight and obese status are considered public health priorities [27]. Currently, the use of pharmacological treatment is becoming pivotal in obesity management [28].

The outcomes of pharmacological treatments for chronic diseases are conditioned by different elements [29]. The efficacy and side effects of antiobesity drugs are critical for the success of these treatments [30]. However, the results of real-world pharmacological interventions are also dependent on treatment adherence [30]. Different factors, such as access to drug information and social considerations, modulate patients’ attitudes toward treatment [31,32]. Therefore, identifying patients’ areas of concern and the sources of information used are relevant for improving the clinical outcomes. Additionally, patients with health behaviors that are frequently disapproved of by society are oftentimes reluctant to disclose to health providers their noncompliance with treatment and medical advice [33]. In this context, the anonymity of social media may provide greater insight into the beliefs, interests, and experiences of patients with regard to antiobesity drugs. Furthermore, family members of the patients, doctors, and health care providers can also participate in the social media community and post their comments related to the pharmacological treatment of obesity. The identification of the needs, concerns, and feelings of the patients related to their treatment may improve their adherence and contribute positively to achieving therapeutic objectives [34].

### Interest in Antiobesity Drugs on Twitter

Our data show that antiobesity drugs are areas of interest within the Twitter community. The attention paid to antiobesity drugs is reflected in the number of tweets posted with content related to these drugs, which was higher than that reported on other medications employed to treat chronic diseases [35,36]. Furthermore, it is also significant that the majority of the posted tweets were related to the medical aspects of antiobesity drugs in contrast to the reported results of other medications in which the interest generated was nonmedical in nature [37]. In addition, the interest of Twitter users was mainly centered on liraglutide and semaglutide, which accumulated nearly 90% of the tweets. Likewise, differences in interest shown by social media users towards drugs with similar clinical indications have been previously observed, for example, in the case of statins [38].

In addition, we also studied users’ areas of interest regarding antiobesity drugs. Our findings show that the one clearly predominant area was drug efficacy, but with different levels of positive consideration being present, as liraglutide, semaglutide, and lorcaserin achieved the highest valuations. With the exception of the tweets related to orlistat, the frequency of references to the side effects of the antiobesity was very low. Considering our study was conducted in the period from September 2019 to October 2019, it is notable that lorcaserin was soon after withdrawn from the market (February 2020) due to its potentially severe side effects [39].

There may be several reasons for these findings. First, differences in the efficacy could explain the different frequencies found between tweets posted about different drugs [30]. However, this factor is unlikely to prove fully conclusive as the results obtained in the clinical trials and metanalysis referencing these drugs do not wholly support the differences observed. Second, there were different patterns of side effects [30]. However, Twitter users have shown little interest in the side effects of the drugs, and it has only been a focus in relation to orlistat and bupropion-naltrexone. Thus, the references to side effects do not seem to explain the differences in interest. Third, it is possible that Twitter users might show a special interest in treatments suppressing appetite. However, this mechanism of action is not only characteristic of glucagon-like peptide-1 inhibitors but also lorcaserin, which only obtained a small number of tweets. The fourth potential factor is availability of scientific information. Liraglutide and semaglutide, the latest approved drugs, have been subject to most of the recent clinical trials involving antiobesity drugs. Furthermore, both drugs have the majority of tweets containing scientific links with content focused mainly on efficacy. Thus, it is possible that recent scientific publications and reports on clinical trials involving liraglutide and semaglutide might in part explain the significant interest toward these drugs within the Twitter community. The fifth reason may involve accessible information in the press and social media. It is known that newly launched drugs or recently approved indications receive enhanced interest from pharmaceutical companies, health providers, and the media [40]. Consequently, the fact that liraglutide and semaglutide have been the most recently approved drugs might explain the special interest towards them.

The dissemination of tweets referencing the 6 different drugs was also heterogenous, with the potential reach and impact of semaglutide clearly being the highest, demonstrating total numbers similar to those of the other 5 drugs combined. This finding could be explained by the fact that semaglutide has been the most recently approved antiobesity drug. In addition, clinical trials concerning semaglutide have been an area of interest for companies and prestigious scientific journals, which have published results in support of the approval of this treatment [41,42].

Furthermore, our data show that most of the tweets were focused on efficacy and rarely mention side effects or discuss issues related to personal adherence to treatment. Thus, there might have been a bias in the information related to these drugs. Moreover, in contrast to previous reports, most of the tweets included a link to sources supporting their content [43]. Interestingly, concerns about the efficacy or tolerability of antiobesity drugs were identified mostly in those tweets not containing a link. The sharing of personal experiences is unlikely to be associated with a link. The analysis of these tweets reveals relevant information for health care providers because many patients that question the efficacy of treatments or abandon their treatments entirely tend to withhold this information from doctors due to feelings of shame or guilt [33]. Indeed, social media has been found to identify side effects not always uncovered via traditional surveys [44]. In semaglutide- and liraglutide-related tweets, most links were scientific, whereas with the rest of the drugs, the majority of links contained a nonscientific source. This may indicate that tweets discussing issues related to semaglutide or liraglutide may be based on medical articles reporting on efficacy. This may thus imply that pharmaceutical companies, scientific journals, and researchers play a key role in Twitter conversations related to obesity medications. Therefore, it is possible that a potential increase in the investigation of the adherence to obesity treatment might increase the dissemination and relevance given by social media users to this pivotal aspect of obesity management.

The important role of social media in generating popular opinion and emotions via information distribution has been established [45], and social media has become a pivotal instrument for sharing knowledge and news [46]. The interest shown by Twitter users in antiobesity drugs support the relevance of social media in the diffusion of medical information. In addition, social media is used to carry out medical interventions, promote preventive health campaigns, and recruit participants for medical research [47,48].

Finally, we studied the sentiment of tweets and found that most drugs obtained a high score. In contrast, bupropion-naltrexone obtained a very low score. This low sentiment toward bupropion-naltrexone may be reflective of the other indications this drug has: smoking cessation or depression. In this regard, it is worth noting the mockery of psychiatric conditions in Twitter [49]. However, this is unlikely to be the only cause of the low score obtained in the sentiment analysis because phentermine-topiramate is frequently prescribed for the treatment of psychiatric conditions and obtained an average score. Thus, it is possible to suggest that this low sentiment is due to the poor effect bupropion has on weight loss [30].

Understanding the public view of the pharmacological treatment of obesity is useful to better appraising the perceived demands for clinical care related to this condition. It could also help designing better promotional health initiatives and awareness strategies that include content of interest to social media users. In addition, this information can facilitate open conversations about a patient’s most common concerns during the medical consultancy. Although this study focused on antiobesity drugs, these results provide relevant information which more than likely can be applied to other pharmacological treatments. The involvement of health institutions in related conversations over social media appears to be desirable given the interest raised by the medical content posted on Twitter.

### Strengths and Limitations

First, the relevance of Twitter as a marker of patient´s voice is a matter of controversy. In addition, tweets do not necessarily reflect the overall experience of patients. Second, regarding the collection of tweets, there is the risk that some were not detected since they might have used different hashtags. However, including brand names and the active pharmaceutical ingredients minimized possible bias related to the choice of hashtags. Third, we did not determine whether the date of FDA approval affected Twitter activity differently in more recent versus less recent medications. Fourth, the codebook design and text analysis involved a degree of subjectivity. Nevertheless, this methodology is consistent with previous medical research studies using Twitter. Although computerized machine learning methods have been tested to automatically identify and classify topics in medical research in social media, we used an analysis strategy based on the raters’ clinical expertise in obesity, which constitutes a qualitative advantage compared to automated strategies [50]. Finally, the inability to verify the precise identity of the majority of Twitter users posting about antiobesity drugs, in addition to a lack of geolocational data, may constitute a limitation in our capacity to interpret results.

### Conclusions

This study demonstrates that Twitter users show an interest in antiobesity pharmacological treatment. The positive consideration of the efficacy of antiobesity drugs accounted for the majority of tweets. In contrast, the side effects of these treatments was only marginally described. Adherence to treatment received little interest from the Twitter community. The nature of the links included in the tweets was heterogenous between the different antiobesity drugs. Thus, this study highlights the opportunity for sharing scientific information, especially that aimed at promoting adherence to pharmacological treatment, which we have detected as being overlooked.

## Abbreviations

FDA: US Food and Drug Administration
JSON: JavaScript Object Notation
